# Evaluation of the reliability and validity of the Medical Outcomes Study sleep scale in patients with painful diabetic peripheral neuropathy during an international clinical trial

**DOI:** 10.1186/1477-7525-6-113

**Published:** 2008-12-17

**Authors:** Muriel Viala-Danten, Susan Martin, Isabelle Guillemin, Ron D Hays

**Affiliations:** 1 Mapi Values, 19 rue de la Villette, 69003 Lyon, France; 2 Pfizer, 2800 Plymouth Road, Ann Arbor, MI 48105, USA; 3 UCLA Department of Medicine/Division of General Internal Medicine & Health Services Research, 911 Broxton Avenue, Room 110, Los Angeles, CA 90024-2801, USA

## Abstract

**Background:**

Sleep is an important element of functioning and well-being. The Medical Outcomes Study Sleep Scale (MOS-Sleep) includes 12 items assessing sleep disturbance, sleep adequacy, somnolence, quantity of sleep, snoring, and awakening short of breath or with a headache. A sleep problems index, grouping items from each of the former domains, is also available. This study evaluates the psychometric properties of MOS-Sleep Scale in a painful diabetic peripheral neuropathic population based on a clinical trial conducted in six countries.

**Methods:**

Clinical data and health-related quality of life data were collected at baseline and after 12 weeks of follow-up. Overall, 396 patients were included in the analysis. Psychometric properties of the MOS-Sleep were assessed in the overall population and per country when the sample size was sufficient. Internal consistency reliability was assessed by Cronbach's alpha; the structure of the instrument was assessed by verifying item convergent and discriminant criteria; construct validity was evaluated by examining the relationships between MOS-Sleep scores and sleep interference and pain scores, and SF-36 scores; effect-sizes were used to assess the MOS-Sleep responsiveness. The study was conducted in compliance with United States Food and Drug Administration regulations for informed consent and protection of patient rights.

**Results:**

Cronbach's alpha ranged from 0.71 to 0.81 for the multi-item dimensions and the sleep problems index. Item convergent and discriminant criteria were satisfied with item-scale correlations for hypothesized dimensions higher than 0.40 and tending to exceed the correlations of items with other dimensions, respectively. Taken individually, German, Polish and English language versions had good internal consistency reliability and dimension structure. Construct validity was supported with lower sleep adequacy score and greater sleep problems index scores associated with measures of sleep interference and pain scores. In addition, correlations between the SF-36 scores and the MOS-Sleep scores were low to moderate, ranging from -0.28 to -0.53. Responsiveness was supported by effect sizes > 0.80 for patients who improved according to the mean sleep interference and pain scores and clinician and patient global impression of change (p < 0.0001).

**Conclusion:**

The MOS-Sleep had good psychometric properties in this painful diabetic peripheral neuropathic population.

**Trial registration:**

As this study was conducted from 2000 to 2002 (i.e., before the filing requirement came out), no trial registration number is available.

## Background

Sleep is an important element of functioning and well-being and is associated with clinical status and general health. Indeed, sleep problems have been found to be associated with depression, anxiety, impaired social functioning, hospitalizations, chronic medical conditions and mortality [1-3]. A number of sleep questionnaires exist that are designed either to measure different aspects of sleep such as quality of sleep, to identify the impact of sleep problems on daily functioning, or to evaluate the impact of sleep disorders on patients' life [4-13].

Painful diabetic peripheral neuropathy (DPN) is one of the most common complications of type I and type II diabetes. Painful DPN may affect sleep, work, social activities and relations, physical mobility, levels of anxiety and depression and energy [14-16], thus leading to the substantial impairments in patients' health-related quality of life (HRQoL) [17,18]. Sleep disturbance is common in chronic pain and is of particular concern in painful DPN as it may influence the progression of type II diabetes [19]. A recent study confirmed the association of painful DPN with sleep impairment [15].

The present study examines perceptions of sleep in an international clinical trial aimed at evaluating the efficacy and safety of the pregabalin, a treatment for pain relief in patients with painful DPN. The Medical Outcomes Study-Sleep Scale (MOS-Sleep) was administered to patients in order to assess the impact of the pregabalin treatment on patients' quality of sleep. We used the data from the international clinical trial to provide information on the psychometric properties of the MOS-Sleep in patients with DPN.

## Methods

### Study population

A total of 512 patients in Germany, Hungary, Poland, Australia, the United Kingdom and South Africa were screened for the clinical trial. Selected patients had to have been diagnosed with type I or II diabetes and with painful, distal, symmetrical, sensorimotor polyneuropathy at least one year prior to inclusion. Three hundred and ninety-seven patients were randomized to medication.

The analysis of the psychometric properties was performed on all subjects who completed at least 50% of the items of the MOS-Sleep at the baseline visit (more than 99% of the sample). The analysis of change over time (responsiveness) of the MOS-Sleep was performed on all subjects who completed at least 50% of the items of the MOS-Sleep at baseline and at termination visits (more than 90% of the sample).

### Study design

The study was a double-blind, randomized, placebo-controlled, multicenter, phase III clinical trial, conducted to evaluate the efficacy and safety of 3 regimens of pregabalin (150, 300, or 600 mg/day) compared to placebo in DPN patients. It was conducted in accordance with the International Conference on Harmonisation Guidelines for Good Clinical Practice (GCPs), the Declaration of Helsinki, and in compliance with United States (US) Food and Drug Administration (FDA) regulations for informed consent and protection of patient rights. The study consisted of a 1-week baseline phase, a 12-week double-blind treatment phase and a 1-week follow-up period. Six visits plus 1 follow-up visit were scheduled during this study [20].

Eligible patients were given a daily pain diary at visit 1 (V1) and had to record pain during the next 7 days of the baseline phase. The diary consists of a single item with an 11-point numeric self-administered rating scale ranging from 0 (no pain) to 10 (worst possible pain). At the end of the baseline phase, a mean of the daily pain scores was calculated; patients with score of 4 or higher were randomized at visit 2 (V2) and started the treatment phase. During the 7 days prior to randomization, patients had to complete the sleep interference diary, in which patients described how their neuropathic pain had interfered with their sleep. It consists of a single-item with an 11-point numerical rating scale, ranging from 0 (pain does not interfere with sleep) to 10 (pain completely interferes with sleep) that allows the calculation of a weekly mean sleep interference score [21-23]. At V2 and at termination visit (V6; 12 weeks after starting medication), patients were asked to complete the MOS-Sleep and the SF-36 questionnaire.

The MOS-Sleep is a 12-item measure developed using patients with chronic illness; it is divided into 6 dimensions evaluating "sleep disturbance," "snoring," "sleep awakening short of breath or with headache," "sleep adequacy," "somnolence," and "quantity of sleep/optimal sleep" [2]. A sleep problems index summarizing information across 9 items of the MOS-Sleep can also be scored. Support for the reliability and validity of the US English version has been reported in the general population, patients with overactive bladder, patients with post-herpetic neuralgia [24,25]; Spanish version properties have been assessed in patients with neuropathic pain [26]. Several language versions (Polish, German and Hungarian) of the instrument have been subsequently developed following rigorous and standardized methodology including 2 forward translations, 1 backward translation, cognitive debriefings and international harmonization [27]; Australian, South African and UK English versions have undergone an adaptation from the original US English version of the MOS-Sleep. The SF-36 v.1 is a generic health survey that includes 36 items measuring 8 multi-item domains [28]: "bodily pain," "general health perception," "mental health," "physical functioning," "role limitation due to emotional problems," "role limitation due to physical health problems," "social functioning" and "vitality." Scores for each of these 8 concepts are transformed linearly to have scores ranging from 0 to 100, higher scores indicating better HRQoL.

Daily pain and sleep interference diaries were collected again at V6. A Clinical Global Impression of Change (CGIC) and a Patient Global Impression of Change (PGIC) were also collected. The CGIC is a clinician-rated instrument that measures change in patient's overall status on a 7-point scale ranging from 1 ("very much improved") to 7 ("very much worse"); the PGIC is a patient-rated instrument that measures change in patient's overall status utilizing the same 7-point scale as above.

### Description and scoring rules of the MOS-Sleep

The item content and the structure of the MOS-Sleep are presented in Table 1. All items of the MOS-Sleep, except item 2, item 10 and item 11, are used to calculate a sleep problems index. The "quantity of sleep" dimension is the average number of hours of sleep per night reported by the patient and the "optimal sleep" is a dichotomized version, that is "yes" when the number of hours of sleep is 7 or 8. The scores of the dimensions and of the sleep problem index were converted to a 0 to 100 scale, with higher scores reflecting more of the attribute implied by the name (e.g. greater sleep disturbance, greater adequacy of sleep).

**Table 1:** Item content of the MOS-Sleep

**Dimensions and Sleep problems index**	**Item #**	**Item contents**
**Sleep disturbance**	07	Trouble falling asleep
	03	Sleep restlessness
	08	Awaken during sleep
	01	Time to fall asleep
**Somnolence**	09	Trouble staying awake
	11	Take naps
	06	Feel drowsy
**Sleep adequacy**	04	Enough sleep, feel rested
	12	Amount sleep needed
**Snoring**	10	Snore during sleep
**Awaken short of breath or headache**	05	Awaken short of breath or headache
**Quantity of sleep/Optimal sleep**	02	Quantity of sleep

**Sleep problems index**	Item # 01, 03, 04, 05, 06, 07, 08, 09, 12	

### Psychometric analysis of the MOS-Sleep

Internal consistency reliability, estimated by Cronbach's alpha coefficient, reflects the extent to which multiple items in a dimension are inter-correlated and form a dimension measuring a same underlying concept [29]. An alpha coefficient of 0.70 or higher is considered a satisfactory level of reliability for group comparisons [30].

Multitrait scaling analysis [31] was used to evaluate the structure of the multi-item dimensions (i.e. "sleep disturbance," "adequacy of sleep" and "somnolence" dimensions and sleep problems index) in order to verify that items measured the concept of their hypothesized dimension. Two criteria were assessed: item convergent criterion (correlation between each item and its own dimension) is met when value is greater than 0.40; item discriminant criterion (extent to which item correlates more highly with the dimension it represents than with other dimensions) states that each item should have a higher correlation with its own dimension than with any of the others.

Construct validity was tested with the following two analyses. The ability of the MOS-Sleep scores to discriminate between groups of subjects according to the severity of the disease was evaluated [32]. The weekly mean sleep interference score and the weekly mean pain score at baseline were used to define groups of patients differing in severity. Since patients had to have pain at baseline to qualify for the present study, the majority of the population tended to be in the most severe of the groups suggested by Zelman et al. [33]. Thus, in the present study, pain groups were defined according to both the cut-points suggested by Zelman and the mean pain score distribution determined from the pain interference diary. As a result, four groupings were defined that contained a balanced distribution of the study population: moderate pain was defined as patients with a score of 3.01 through 6.00, and three categories of severe pain were defined as patients with a score of 6.01 through 7.00, patients with a score of 7.01 through 8.00, and patients with a score of 8.01 through 10.00. As no cut-points were published for the sleep interference score, groups similar to those used for the pain score were defined: no or mild sleep interference was 0 through 3.00; moderate sleep interference was 3.01 through 6.00; and two severe sleep categories were defined as those with a score of 6.01 through 7.00, and those with a score of 7.01 through 10.00. As HRQoL data tend to be non-normally distributed, Kruskal-Wallis and Chi-square non-parametric tests were computed to compare MOS-Sleep scores by the different severity groups.

Validity was also assessed by examining Spearman rank-order correlations between the MOS-Sleep scores and the SF-36 scores. Based on previous work [34], we hypothesized that the highest correlations would be between sleep and mental health dimensions of the SF-36; moderate correlations were expected between all SF-36 scores and the MOS-Sleep scores, except for the MOS-Sleep "snoring" score for which low correlations were expected.

Responsiveness to change was evaluated for the MOS-Sleep scores for groups of patients based on their change in health status over 12 weeks (between V2 and V6). Using the CGIC and PGIC, subgroups of 'much improved,' 'improved,' 'stable' and 'worsened' patients were defined. In the absence of published thresholds, the following groups of patients were defined based on the distribution of the changes in both the weekly sleep interference and pain diaries mean scores: 'much improved' (-10 ≤ mean sleep/pain score ≤ -4); 'improved' (-4 < mean sleep/pain score ≤ -1); 'stable' (-1 < mean sleep/pain score < 1); and 'worsened' (1 ≤ mean sleep/pain score ≤ 10). Effect Size (ES) and the Standardized Response Mean (SRM) were calculated, with the following values used for the interpretation of ES and SRM: ES and SRM = 0.20, small change; ES and SRM = 0.50, moderate change; ES and SRM = 0.80, large change [35,36]. The responsiveness analysis was performed on the subjects for whom the MOS-Sleep was completed at baseline and at termination visit and is considered assessable (i.e. at least 50% of the items were completed).

Statistical analyses were performed on the overall population and for the five countries of the study separately.

Main analyses were performed using SAS software (Statistical Analysis System, version 8.02) for Windows. Multitrait analyses were performed using the MAP-R program. The threshold for statistical significance was set up at 5%.

## Results

### Description of the population at baseline

Three hundred and ninety-six patients had an assessable questionnaire (i.e. at least 50% of the items completed) at baseline visit and were included in the psychometric analyses. Socio-demographic and clinical characteristics of the sample are summarized in Table 2. Seventy-seven patients were from Australia, 66 from Germany, 34 from Hungary, 166 from Poland, 32 from South Africa and 21 from the United Kingdom. The majority of patients (55%) were men, ranging from 38% in Hungary to 77% in Australia. Patients' mean age was 59 (standard deviation (STD) = 11 years), ranging from 55 (STD = 12 years) in Poland to 63 (STD = 10 years) in Australia. Patients in Poland, South Africa and the United Kingdom were slightly younger than in Australia, Germany and Hungary.

**Table 2:** Description of the population at baseline (N = 396)

**Population characteristics**		
**Socio-demographic characteristics**		
Age (years; mean ± STD)		59 ± 11
Gender	Female	45%
	Male	55%
Race	White	96%
	Black	1%
	Asian or Pacific	2%
	Other	2%
Country	Australia	19%
	Germany	17%
	Hungary	9%
	Poland	42%
	South Africa	8%
	United Kingdom	5%

**Clinical parameters**		**Mean score **± **STD**
Mean sleep interference score		5.6 ± 2.2
Mean pain score		6.4 ± 1.4

**MOS-Sleep dimension scores**		
Awaken Short of Breath		24.3 ± 27.2
Sleep disturbance		54.9 ± 26.3
Quantity of sleep		6.0 ± 1.6
Snoring		40.2 ± 33.3
Somnolence		42.0 ± 24.2
Sleep Problems Index		48.8 ± 19.6
Sleep adequacy		43.5 ± 28.7
Optimal sleep (yes; %)		34

STD: Standard Deviation

Mean sleep interference scores ranged from 5.37 (STD = 2.16) in Poland to 6.49 (STD = 2.45) in South Africa at baseline. The mean sleep interference score across countries was 5.59 (STD = 2.17). Mean pain scores ranged from 6.14 (STD = 1.44) in Germany to 7.48 (STD = 1.52) in South Africa. The mean pain score across countries was 6.42 (STD = 1.44).

### Item missing data for the MOS-Sleep

Percentage of item missing data ranged from 0.0% in South Africa and the United Kingdom to 1.5% in Germany at baseline (V1) and from 0.0% in Hungary to 1.6% in the United Kingdom at termination visit (V6).

### Psychometric properties of the MOS-Sleep

#### Internal consistency reliability

Cronbach's alphas for the MOS-Sleep dimensions ranged from 0.71 to 0.81 (Table 3) for all countries combined. The sleep problems index and "sleep disturbance" dimensions exceeded the standard criteria for reliability (i.e. Cronbach's alpha ≥ 0.70) in each of the countries, with Cronbach's alphas ranging from 0.76 to 0.90 and 0.77 to 0.82, respectively. The threshold of 0.70 was reached for the "somnolence" dimension for all the language versions, except the German (0.61), Hungarian and South African (0.67) ones. The "sleep adequacy" dimension reached the threshold value only for German and Polish versions.

**Table 3:** Cronbach's alpha and item convergent and discriminant criteria of the MOS-Sleep multi-item scores (N = 381)

**Multi-item scores**	**No of items**	**Cronbach's alpha**	**Range of item-scale correlations**	**% of items meeting the convergent criterion**	**% of items meeting the discriminant criterion**
**Sleep disturbance**	4	0.80	0.51–0.76	100%	100%
**Somnolence**	3	0.74	0.47–0.63	100%	100%
**Sleep adequacy**	2	0.71	0.55–0.55	100%	100%
**Sleep problems index**	9	0.81	0.34–0.62	78% (all except items 5 and 9)	*NA*

N = 381 patients with all items completed are included in this analysis; NA = Not Applicable

#### Multitrait Scaling Analysis

Items in the "sleep disturbance," "somnolence," and "sleep adequacy" dimensions of the MOS-Sleep had item-scale correlations ranging from 0.47 to 0.76 (Table 3). Items of the sleep problems index had correlations ranging from 0.34 to 0.62 (only items 5 and 9 did not reach the threshold of 0.40). All items had a higher correlation with their own dimensions than with the others. Per language version, the majority of items had item-scale correlations higher than 0.40 for their hypothesized dimension, except for items 4 and 12 of the "sleep adequacy" dimension in the Australian and Hungarian language versions (correlation = 0.37 and 0.34, respectively), item 11 of the "somnolence" dimension in the German, Hungarian and South African language versions (correlation = 0.28, 0.38, 0.34, respectively), and item 3 of the "disturbance" dimension in the Hungarian language version (correlation = 0.34). Item discrimination criterion across dimensions was satisfied, except for item 12 of the "sleep adequacy" dimension of the Australian, Hungarian and UK English versions, and item 4 of the South African, item 6 of the "somnolence" dimension of the German and South African and item 3 of the "sleep disturbance" dimension of the Hungarian version.

#### Construct validity

**Figure 1:**
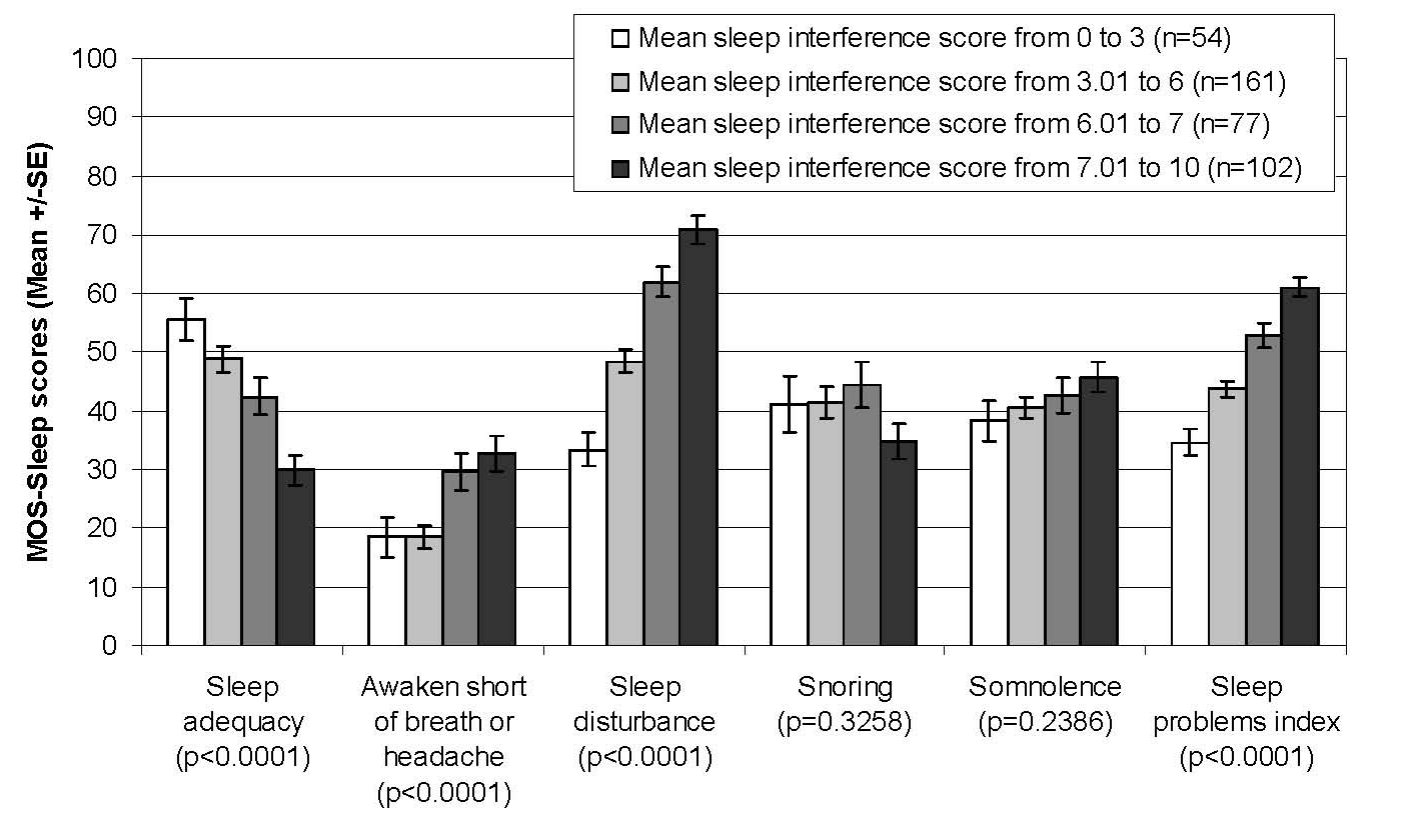
**MOS-Sleep scores according to the mean sleep interference score at baseline; SE: Standard Error; p: Kruskal-Wallis p-value for between-group comparison**.

**Figure 2:**
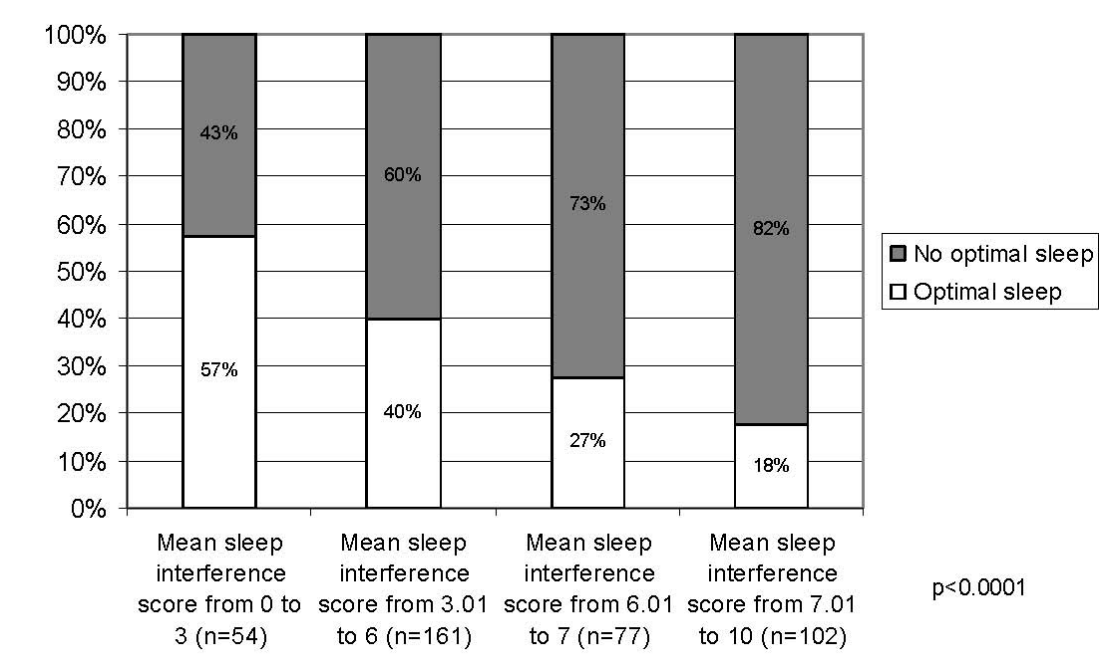
**Percentage of patients with optimal sleep according to the mean sleep interference score at baseline; p: chi-square p-value for between-group comparison**.

Figure 1 displays the mean scores of the MOS-Sleep dimensions for each severity group as defined with the mean sleep interference score at baseline. Most of the MOS-Sleep dimensions were able to discriminate between patients with different levels of severity of sleep interference: the higher the mean sleep interference scores, the lower the "sleep adequacy" mean score and the higher the mean scores of the dimensions "awaken short of breath or with headache", "sleep disturbance" and "somnolence", and the sleep problems index; only the "snoring" dimension did not follow any of these trends. The difference between severity groups was statistically significant (p < 0.0001) for all dimensions, except "snoring" (p = 0.3258) and "somnolence" (p = 0.2386), and for the sleep problems index (p < 0.0001). Higher mean sleep interference score was associated with lower percentage of patients with optimal sleep (i.e., 7 to 8 hours sleep per night) (Figure 2). The difference between the groups of severity was highly significant (p < 0.0001).

**Figure 3:**
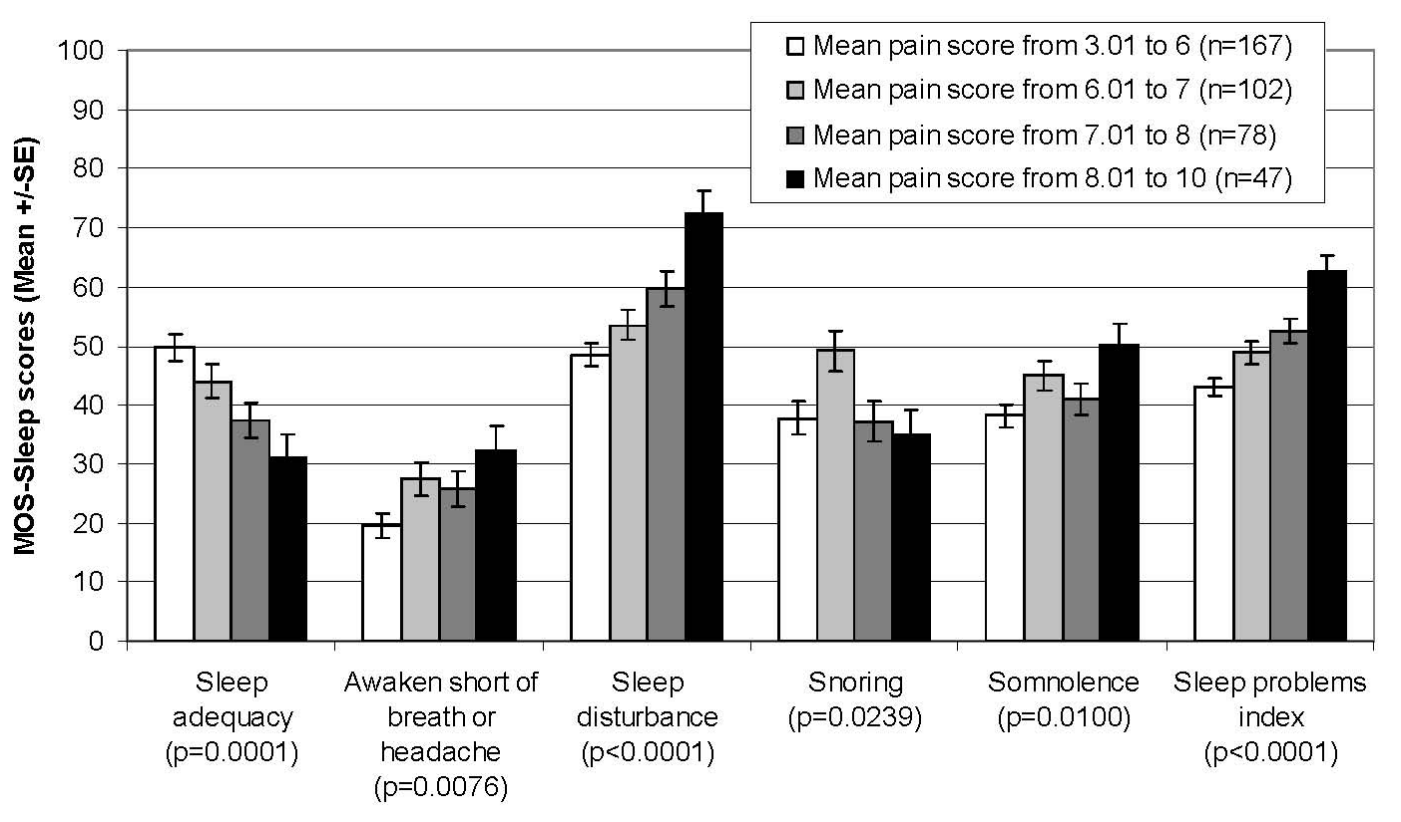
**MOS-Sleep scores according to the mean pain score at baseline; SE: Standard Error; p: Kruskal-Wallis p-value for between-group comparison**.

**Figure 4:**
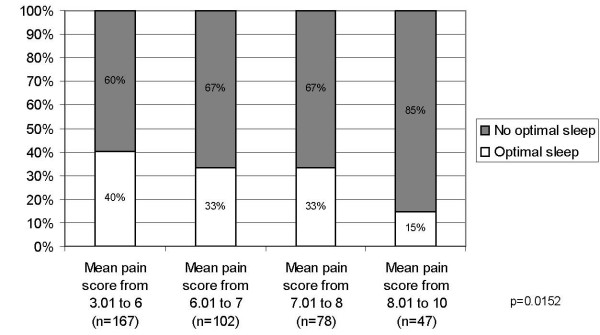
**Percentage of patients with optimal sleep according to the mean pain score at baseline; p: chi-square p-value for between-group comparison**.

Similarly, the MOS-Sleep dimensions discriminated between patients with different level of pain: the higher the mean pain score at baseline, the higher the mean scores of the dimensions "awaken short of breath or headache," "somnolence" and "sleep disturbance," and of the sleep problems index; the lower the mean score of the "sleep adequacy" dimension (Figure 3). While the relation between pain severity and mean score was monotonic for the "sleep disturbance," the "sleep adequacy" dimensions and for the sleep problems index, the trend was less clear for "awaken short of breath or with headache," "snoring" and "somnolence" dimensions (Figure 3). The difference between groups of pain severity was significant for all the dimensions as well as for the sleep problems index (p < 0.020). There was also a significant association (p = 0.015) between the "optimal sleep" score and the mean pain score, with percentage of patients with "no optimal sleep" score increasing as pain severity increased, as illustrated in Figure 4.

Spearman correlations coefficients between the MOS-Sleep scores and the SF-36 scores at baseline are summarized in Table 4. Correlations of 0.40 or higher (absolute value) were found between the sleep problems index of the MOS-Sleep and "bodily pain," "mental health," "physical functioning," "social functioning" and "vitality" scores of the SF-36, between "sleep adequacy" and "vitality" scores and between "sleep disturbance" and "bodily pain." The highest correlation (-0.53) was found between the sleep problems index and the SF-36 "vitality" score. Correlations in the 0.30 to 0.40 range were found between "sleep disturbance," "sleep adequacy" and the sleep problems index and the SF-36 "bodily pain," "general health perceptions," "mental health," "physical functioning," "role limitations due to physical health problems," "social functioning" and "vitality" scores. The "snoring" score was not correlated with any of the SF-36 scores.

**Table 4:** Spearman correlation coefficients between MOS-Sleep scores and SF-36 scores at baseline (N = 396)

**SF-36 scores**	**MOS-Sleep scores**						
	
	**Sleep disturbance**	**Somnolence**	**Sleep adequacy**	**Snoring**	**Short of breath or headache**	**Quantity of sleep**	**Sleep problems index**
**Bodily pain**	-0.41**	-0.27**	0.32**	0.03	-0.27**	0.23**	-0.47**
**General health**	-0.22**	-0.17*	0.27**	-0.06	-0.27**	0.04	-0.31**
**Mental health**	-0.32**	-0.16*	0.36**	0.03	-0.26**	0.13*	-0.42**
**Physical functioning**	-0.34**	-0.28**	0.25**	-0.04	-0.27**	0.16*	-0.40**
**Role-emotional**	-0.19**	-0.15*	0.23**	0.04	-0.20**	0.07	-0.28**
**Role-physical**	-0.27**	-0.19*	0.30**	-0.02	-0.22**	0.12*	-0.35**
**Social functioning**	-0.31**	-0.25**	0.36**	-0.06	-0.25**	0.08	-0.43**
**Vitality**	-0.39**	-0.30**	0.44**	-0.02	-0.28**	0.19**	-0.53**

Note: * p < 0.05; **p < 0.0001

#### Responsiveness

The change in score on the sleep problems index was evaluated according to change in mean sleep interference score, change in mean pain score, CGIC and PGIC between V2 and V6 (Table 5). Similar data were observed for the other MOS-Sleep dimensions. Mean changes in the sleep problems index score differed according to the changes in health status over the 12-week study: change in the sleep problems index was greater as pain and sleep of patients improved; a similar trend was observed with patients' and clinicians' global impression of change. The changes in the sleep problems index were statistically different between groups defined according to the changes in sleep interference score, changes in pain score, clinician and patient global impression of change (p < 0.0001).

**Table 5:** Distribution of the change in the MOS sleep problems index according to the change in health status (N = 356)

		**Change in scores of the** **MOS sleep problems index**					
		**N**	**Mean**	**STD**	**ES**	**SRM**	**p(change = 0)**

**Change in mean sleep interference score** **p(KW) < 0.0001**	Much improved (-10 to -4)	77	-25.90	20.41	-1.42	-1.33	<0.0001
	Minimally improved (-4 to -1)	171	-12.00	17.54	-0.68	-0.70	<0.0001
	No change (-1 to +1)	83	-7.01	18.73	-0.27	-0.31	0.0005
	Worse (+1 to +10)	25	-3.12	18.97	-0.22	-0.22	0.3603

**Change in mean pain score** **p(KW) < 0.0001**	Much improved (-10 to -4)	76	-22.91	21.07	-1.20	-1.16	<0.0001
	Minimally improved (-4 to -1)	145	-14.37	19.49	-0.75	-0.74	<0.0001
	No change (-1 to +1)	119	-5.71	16.99	-0.28	-0.32	0.0003
	Worse (+1 to +10)	16	-12.63	15.66	-0.64	-0.81	0.0065

**Clinician global impression of change** **p(KW) < 0.0001**	Very much improved	30	-28.27	20.68	-1.41	-1.41	<0.0001
	Much improved	127	-20.07	18.78	-1.13	-1.11	<0.0001
	Minimally improved	111	-10.58	18.42	-0.61	-0.60	<0.0001
	No change	74	-1.19	15.84	-0.01	-0.01	0.3380
	Worse	14	-3.36	11.22	-0.11	-0.25	0.2896

**Patient global impression of change** **p(KW) < 0.0001**	Very much improved	30	-26.73	23.07	-1.27	-1.24	<0.0001
	Much improved	123	-21.38	18.11	-1.19	-1.22	<0.0001
	Minimally improved	114	-10.25	18.66	-0.60	-0.56	<0.0001
	No change	67	-1.73	13.42	-0.06	-0.10	0.1838
	Worse	22	0.45	16.06	0.11	0.18	0.8336

N: number of patients per group; STD: standard deviation; ES: effect-size; SRM: standard response mean; p(change = 0): Wilcoxon signed-rank test p-value; p(KW): Kruskal-Wallis p-value

ES and SRM were > 0.80 for the 'much improved' group of patients, between 0.50 and 0.80 for the 'minimally improved' group of patients, and between 0.20 and 0.50 for the 'no change' and 'worse' groups whatever the criterion used to define these groups. Changes in the sleep problems index score were highly statistically different from 0 for all the groups of improved ('very much', 'much' and 'minimally') patients based on the mean sleep interference score, the pain score, and the clinician and patient global impression of change (p < 0.0001) (Table 5). Stable patients, as defined by no change in the mean sleep interference score or no change in the mean pain score, also showed a significant (but lower than for the 'improved' group) change in MOS sleep problems index score (p = 0.0005 and 0.0003, respectively), whereas stable patients as defined by the CGIC and the PGIC showed no statistically significant change in MOS sleep problems index score. Changes in the sleep problems index score of the 'worsened' patients defined on the mean sleep interference score, the CGIC and the PGIC were not statistically different from 0 (Table 5).

## Discussion

The objective of this study was to provide information on the psychometric properties of the MOS-Sleep in a DPN population. The instrument is a 12-item questionnaire developed to evaluate patient reported sleep outcomes in terms of "sleep disturbance," "sleep adequacy," "somnolence," "quantity of sleep," "snoring" and "awaken short of breath or with a headache."

The MOS-Sleep was included in an international clinical trial conducted in Germany, Hungary, Poland, Australia, United Kingdom and South Africa; the respective language version of the instrument was administered in each of the countries. Psychometric properties were assessed on the pooled data across the 6 countries. When the sample sizes permitted it, the psychometric properties of the MOS-Sleep were evaluated for each of the translated versions in each country.

A total of 396 patients with DPN participated in the study, representative of differing socio-demographics (ethnicity, gender, age) and clinical characteristics. Item completion rates were similar for each language version of the MOS-Sleep, suggesting good acceptability of the questionnaire by patients across the 6 countries.

Based on the results of the psychometric evaluation, the different language versions of the MOS-Sleep displayed adequate and generally comparable reliability and validity. The responsiveness of the tool was also demonstrated on the pooled population.

The robustness of the structure of the "sleep disturbance" and "somnolence" multi-item dimensions and the sleep problems index of the MOS-Sleep versions was demonstrated, whether tested on the pooled population or on the country-based populations. Dimensions showed satisfactory to excellent item convergent validity in all versions; only items 4 and 12 of the "sleep adequacy" dimension for the Australian and Hungarian versions, item 11 of the "somnolence" dimension for the German, Hungarian and South African versions, and item 3 of the "disturbance" dimension for the Hungarian version showed poorer results when compared to the other versions. This might be related to translation difficulties in these language versions. Most of the items satisfied the requirements for item discrimination across dimensions, that is items were more strongly correlated with their hypothesized dimensions than with the other dimensions of the instrument.

Internal consistency reliability of the sleep problems index, "sleep disturbance" and "sleep somnolence" showed good to excellent results whether the populations of the 6 countries were pooled or analyzed separately. Only the results for the "sleep adequacy" dimension were the least satisfactory, with Cronbach's alpha reaching the recommended threshold value of 0.70 only for the German and Polish language versions. This is likely to be explained by "sleep adequacy" is a 2-item scale, as dimensions with only 2 or 3 items are susceptible to have lower Cronbach's alpha than dimensions with a greater number of items. Because of the study design that lasted 12 weeks, test-retest reliability of the MOS-Sleep could not be evaluated in the trial, as this property requires a short enough time between the two points of data collection so that there is minimal or no change on the attribute being measured. So far, there is no published test-retest reliability assessment for the MOS-Sleep.

Correlations between the MOS-Sleep and SF-36 dimensions provided evidence for construct validity. The sleep problems index was moderately correlated (0.40 ≤ r < 0.70) with most of the SF-36 dimension scores, highlighting the relationship between sleep and other aspects of patients' health-related quality of life. This relationship is in agreement with previous work in general and chronic disease populations, which demonstrated that sleep disorders adversely affected general health as well as functional status, work performance, mood and everyday functioning [1,37-40]. It also suggested that sleep has an impact not only on physical but also on mental and social functioning, confirming previous reports in DPN patients using the SF-12 tool or the Nottingham Health Profile [1,16]. As hypothesized, the highest overall correlations were observed between the "sleep disturbance" and the "sleep adequacy" scores and the SF-36 scores, whereas the "snoring" dimension was the most weakly correlated with the SF-36 scores. The sleep problems index shared the strongest correlation (-0.53) with the SF-36 "vitality" dimension.

The MOS-Sleep dimensions were able to discriminate between patients with various levels of sleep interference and pain severity, with greater impairment observed for more severe patients thus providing further support for the validity of the instrument. However the categories used to classify patient severity could be discussed. Indeed because of the study design (patients had to have pain at baseline), the majority of our trial population was classified in the "severe" group (> 7) as defined by Zelman et al. [33]. Therefore, in order to better reflect the distribution of the pain severity of our population, grouping was performed based on slightly different cut-points which are specific to our population.

The responsiveness of the MOS-Sleep observed here was consistent with previous studies performed in patients with neuropathic pain [24,26]. The sleep problems index score proved to be highly responsive to clinical changes, whether sleep interference, pain, patient or clinician global impressions were used to define improvement, stability or worsening over the 12-weeks period of study. Overall, the magnitude of change as measured by ES for the sleep problems index was the largest for patients who improved over the 12 weeks, and decreased as patients' health status became stable or worsened based on sleep interference, pain, patient or clinician global impression of change. The changes observed were statistically different from zero for all the improved subgroups. Surprisingly, these changes in scores were also significantly different from zero for patients classified as stable according to the change in mean sleep interference and mean pain scores. These unexpected findings could be explained by the fact that as patients are being treated, most of the patients classified as stable are actually minimally improved. It also suggests that pain and the other factors, taken individually, can not be the only determinants of the effect observed on patients' sleep. 

Results and observations that could be drawn from the pooled language versions should be taken with caution. Even if some results showed the reliability and validity of the translated versions, they do not allow the cross-cultural validity of the MOS-Sleep to be fully addressed. Cross-cultural validity is a very complex notion including several levels of equivalence that should be checked and fulfilled in order to conclude that the language versions of an instrument are cross-culturally equivalent [41]. In order to address the cross-cultural equivalence of the different versions of the MOS-Sleep, further analyses would be necessary, requiring a larger number of patients than what we had in the present study.

## Conclusion

The MOS-Sleep had good psychometric properties in this study's DPN population, which vary from one language version to an other. While Australian, Hungarian and South African versions of the MOS-Sleep showed limited psychometric properties when taken individually in the specific conditions of the clinical trial, German, Polish and English language versions display good internal consistency reliability and structure.

## Competing interests

This research was sponsored by Pfizer Inc. MVD and IG are employees of Mapi Values, who were paid consultants to Pfizer in connection with the development of the manuscript. SM was a Pfizer employee at the time of the study, and owned Pfizer stock. RDH, was supported in part by the UCLA Resource Center for Minority Aging Research/Center for Health Improvement in Minority Elderly (RCMAR/CHIME), NIH/NIA Grant Award Number P30AG021684, and the UCLA/DREW Project EXPORT, NCMHD, P20MD000148 and P20MD000182.

## Authors' contributions

MVD performed the statistical analysis and interpretation of findings, and reviewed the manuscript. SM was responsible for the study design, study implementation, review and input into the manuscript. IG wrote the manuscript. As the developer of the MOS-Sleep, RDH helped in the interpretation of findings and reviewed the manuscript.
